# Goals of care discussions among critically Ill patients on vasopressor treatment

**DOI:** 10.1371/journal.pone.0324482

**Published:** 2025-05-28

**Authors:** Walid A. Alkeridy, Khalid M. Alayed, Shadan AlMuhaidib, Mohammed Khalid Alageel, Sarah Ahmed Alqahtani, Shatha Bin Dher, Abdulaziz M Almotairi, Claire Ann Lim, Musa F. Alzahrani

**Affiliations:** 1 Department of Medicine, College of Medicine, King Saud University, Riyadh, Saudi Arabia; 2 Department of Medicine, Division of Geriatric Medicine, University of British Columbia, Vancouver, British Columbia, Canada; 3 General Administration of Home Health Care, Therapeutic Affairs Deputyship, Riyadh, Saudi Arabia; 4 Independent Researcher, Riyadh, Saudi Arabia; 5 Department of Emergency Medicine, College of Medicine, King Saud University, Riyadh, Saudi Arabia; 6 Department of Emergency Medicine, University of British Columbia, Vancouver, Canada; 7 College of Medicine, King Saud University, Riyadh, Saudi Arabia; 8 Department of Medical Rehabilitation, King Saud Medical City, Riyadh, Saudi Arabia; 9 Oncology Center, King Saud University Medical City, King Saud University, Riyadh, Saudi Arabia; Government Villupuram Medical College and Hospital, INDIA

## Abstract

**Background:**

Goals of care (GOC) discussions are essential for aligning medical management with the values of critically ill patients, particularly those requiring vasopressors, such as dopamine. To evaluate GOC discussions in terms of prevalence, factors associated with documentation, and impact on survival among critically ill patients requiring vasopressors for hemodynamic support.

**Methods:**

We conducted a retrospective cohort study at a tertiary healthcare facility in Riyadh, Saudi Arabia, focusing on patients admitted to intensive care units (ICUs) and internal medicine (IM) wards. The study included adult in patients who received dopamine during their hospital stay. Factors associated with GOC documentation were identified using logistic regression analysis. The 30-day and 1-year survival rates according to GOC discussions were analyzed using Kaplan–Meier survival curves, which were compared using the log-rank test.

**Results:**

Of 301 patients, 56.8% were men and 64.8% were aged ≥60 years. GOC discussions were documented in 61.8% of patients and were more frequent among older patients (≥60 years) than among younger patients (73.1% vs. 51.3%, p < 0.001) and in those with higher APACHE II scores (median 21.0 vs. 18.0, p = 0.001). Multivariable analysis identified age ≥ 60 years as independent precipitating factor of GOC discussions (odds ratio 2.41, 95% confidence interval 1.34–4.32, p = 0.003). The study found significantly lower survival rates at both 30 days and 1 year among patients who had documented GOC discussions.

**Conclusions:**

GOCs were more prevalent among critically ill older patients. The study found significantly higher mortality rates at both 30 days and 1 year among patients who had documented GOC discussions. These findings highlight the need for institutional strategies to integrate GOC discussions into routine care and address their potential implications on patient outcomes.

## Introduction

Involvement of patients in medical decisions is a fundamental aspect of healthcare delivery, contributing to improved patient satisfaction, better clinical outcomes, and alignment of medical interventions with patient values and preferences [1–3]. The concept of goals of care (GOC) discussion entails a crucial dialog among healthcare providers, patients, and families to align medical treatment with the values, preferences, and prognosis of critically ill patients [4]. GOC discussions are structured compassionate conversations that aim to understand and incorporate patients’ wishes into their medical treatment plan. The reported prevalence of conversations regarding advanced care ranges from 10.1% to 86.0% in the United States [5–8] and is 34.4% in Australia [9]. In the United States, GOC conversations are often delayed or limited, but when established, these practices have been associated with improved transition to comfort-focused care, increased alignment of care with patient values and reduced non-beneficial interventions [5–8]. Similarly, in Australia, patients with life-threatening illnesses exhibited high one-year mortality rates, yet GOC practices remained limited—highlighting missed opportunities to align care more effectively with patient preferences. However, in the Middle East and North Africa region, explicit studies on the prevalence of GOC discussions among critically ill patients are lacking.

The relevance of GOC discussions cannot be overstated among older adults with multiple comorbidities [10]. Several older adults with acute illness require hemodynamic support to manage shock comprehensively [11]. Such interventions are commonly delivered in intensive care units (ICUs) by skilled healthcare providers and require frequent monitoring and adjustments [12]. Considering the high risk of adverse outcomes among critically ill patients, urgent effective discussions with patients to arrive at complex decisions on escalation or de-escalation of care are needed [10,13]. Therefore, understanding the short-term (30-day) and long-term (1-year) survival outcomes in the context of GOC discussions is important, because it would enable clinicians to effectively align medical interventions with patient-centered care [14–16].

In areas with distinct cultural and religious contexts, understanding and implementing GOC practices provide a unique framework. With its deep roots in Islamic values, Saudi Arabia exemplifies such a setting. Islamic bioethics, which is a cornerstone of medical decision-making in the region, acknowledges the concept of medical futility, particularly when continued treatment offers no meaningful benefit to the patient. [17]; However, this perspective may differ from Western bioethical frameworks, which tend to place greater emphasis on individual autonomy in end-of-life decision-making. [18]. In Islamic bioethics, interventions that are deemed excessive and fail to contribute to meaningful patient outcomes are discouraged, emphasizing the importance of measured and purposeful care [19]. Furthermore, in Islamic traditions, the concepts of prioritizing the prevention of harm (*la darar wa la dirar*) and communal benefit (*maslahah*) are integrated core ethical principles, which are essentially considered in the decisions of withdrawing or withholding life-sustaining interventions [17,20]. Such a pattern includes a collective decision-making paradigm, involving family members and probably religious authorities; hence, the structure of GOC discussions in Saudi Arabia and other Islamic countries are uniquely affected.

Despite this conceptual alignment, there is a lack of literature on the patterns, predictors, and outcomes of GOC documentation from a Saudi perspective. Existing research has not adequately explored the interplay among cultural, religious, and systemic factors that influence the adoption of GOC practices. Addressing this gap is crucial for understanding how local approaches can be optimized to ensure ethical, culturally sensitive, and patient-centered care. Further research is needed to identify the key factors supporting the integration of GOC documentation into clinical practice and to evaluate its impact on healthcare delivery and patient outcomes in the region [21].

The use of peripheral vasopressors for shock management has gained wider acceptance to facilitate timely intervention in hemodynamically-unstable patients [22]. Among hemodynamically unstable patients, peripheral vasopressors can be used for early resuscitation, as a temporizing measure to stabilize the patient before transfer to a critical care setting, or as a bridge for central venous catheter (CVC) placement in shock management, particularly when immediate central access is not feasible [22]. Although it has previously been shown that vasopressors may reduce the need for CVC during the entire course of treatment, the most recent Surviving Sepsis Campaign guidelines recommended peripheral initiation just to avoid delays in vasopressor therapy [23]. Despite recent evidence on its inferior outcomes compared with other vasoactive agents for shock [24–26], dopamine remains frequently prescribed and is often perceived as safe for peripheral administration for critically ill patients [27]. Although dopamine is usually initiated in critically ill patients at high risk of adverse outcomes, GOC discussions are often missing or undocumented in this context, and their association with survival outcomes is unclear. Moreover, the lack of comprehensive discussions with patients or their families regarding the potential clinical outcomes raises important ethical concerns. Therefore, the GOC discussion patterns and possible outcomes, including survival rates, should be explored in critically ill patients. Moreover, the differences in the outcomes of critically ill patients between the ICU and IM ward settings are unknown. Addressing these gaps can provide insights into the current patterns of GOC discussion and potential strategies to improve care for patients with critical conditions [28].

This study aimed to evaluate the prevalence and factors associated with GOC discussions among critically ill patients treated with dopamine and to assess the association between documented GOC discussions and survival outcomes, including 30-day and 1-year mortality rates.

## Methods

### Study design, study population, and eligibility criteria

This retrospective study was conducted at King Khalid University Hospital (KKUH) in Riyadh, Saudi Arabia. The primary objective of this study was to identify clinical and demographic predictors associated with the documentation of GOC discussions among hospitalized adults who received dopamine for hemodynamic instability. GOC documentation was treated as the primary outcome in logistic regression analysis. A secondary objective was to compare 30-day and one-year survival between participants with and without documented GOC discussions. A matched cohort of ICU patients receiving dopamine was included to assess differences in GOC practices across care settings, rather than to evaluate mortality differences between groups. We included adult inpatients (aged ≥18 years) in the ICU and noncritical care (IM wards) settings who needed dopamine as a vasopressor for hemodynamic instability. At KKUH, dopamine is the only vasopressor permitted for peripheral administration in hemodynamically unstable patients in the IM wards. The eligibility criteria included patients who received dopamine, were admitted to the IM wards for acute medical conditions, and were considered non-ICU candidates during their current or prior admissions, as documented in the ICU notes. The exclusion criteria included patients who were previously admitted to the ICU but were later transferred to the IM ward, as well as those classified as non-ICU candidates because of ICU admission refusal by either the patients or their families. We screened a total of 136,883 hospital admission records between January 1, 2019, and December 31, 2022, to identify patients who received dopamine. Among these, 348 patients were found to have received dopamine. After applying inclusion and exclusion criteria, 301 patients were included in the final analysis. The 47 excluded patients did not meet one or more eligibility criteria or were admitted in other hospital settings (e.g., transferred to ICU during their admission). The follow-up period was extended to December 31, 2023, to facilitate a comprehensive evaluation of 30-day and 1-year survival outcomes. All participants were followed from the date of initial admission, and those lost to follow-up before completing one year were censored at their last recorded survival date. No participants were excluded due to missing data, as all essential clinical variables—including those required for APACHE II scoring—were consistently available in the medical records. The predefined clinical variables included age, which was categorized into <60 vs. ≥ 60 years old; cardiovascular disease; malignancy; dementia; history of previous stroke; acute renal failure; Acute Physiology and Chronic Health Evaluation (APACHE II) score; Glasgow Coma Scale (GCS); and dopamine dose in µg/kg/min, which was categorized as low (<5), moderate (5–10), or high (>10). Overall survival was defined as the time from admission to death from all causes, censored at 30 days and 1 year.

### Data collection

Data were retrospectively collected using standardized forms to ensure accuracy and consistency. GOC documentation was identified through a comprehensive and standardized review of medical records. Documentation explicitly labeled “GOC” or notes reporting GOC conversation components, including preferences, care goals, and medical interventions, were considered valid. For patients with do-not-resuscitate (DNR) orders, the availability of completed DNR forms, which included dedicated GOC sections, was also considered evidence of GOC discussions. A trained data collector systematically reviewed all electronic and paper-based medical records to ensure accuracy and consistency, focusing on documentation explicitly labeled as “Goals-of-Care discussion” or containing the core elements of such discussions. This review involved an analysis of documented DNR forms, which at our institution feature specific sections that reflect GOC discussions. In instances of ambiguous or unclear documentation, a trained internist reviewed the records for adjudication. Remaining discrepancies were resolved through consensus with a third reviewer, an experienced hospitalist, to ensure consistency and reduce misclassification bias.

### Ethics

Ethical approval was obtained from the Institutional Review Board (IRB) of KKUH (Approval No. E-23–7681) on March 14, 2023, and data were accessed on May 14, 2023. The IRB granted a waiver of informed written consent due to the retrospective nature of the study. Data were exclusively retrieved from medical records, ensuring participant anonymity, and were collected solely for research purposes in compliance with ethical guidelines. This study was conducted in accordance with the principles of the Declaration of Helsinki on ethical standards for medical research involving human subjects.

### Statistical analysis

All statistical analyses were conducted using IBM SPSS Statistics, Version 29.0 (IBM Corp., 2023. Given the retrospective design of the study, no formal a priori sample size calculation was required. However, the final sample of 301 participants exceeded commonly accepted thresholds for multivariable logistic regression analysis [29]. Categorical variables were summarized as frequencies and percentages and compared using chi-square test or Fisher’s exact test, as appropriate. Continuous variables were tested for normality using Shapiro–Wilk test. All variables were nonnormally distributed and were reported as median with interquartile range (IQR) and range. The associations between clinically relevant variables and GOC discussions were explored using logistic regression analyses. Statistical significance was defined as a P-value <0.05. Variables identified to be significant in the univariable models were then included in the multivariable logistic regression to determine the independent factors associated with GOC discussions. Results were reported as odds ratio (OR) with 95% confidence intervals (CI) and P-value. Kaplan–Meier survival curves were used to analyze 30-day and 1-year survival after adjusting for patient GOC discussion status, and the results were compared using the log-rank test.

## Results

### Demographic and clinical characteristics

This study included 301 patients with a median age of 66 years (IQR = 55–79 years, range 18–104 years) (Table 1). Most patients were aged ≥60 years (n = 195, 64.8%). There was a higher proportion of men than of women (n = 171, 56.8% vs. n = 130, 43.2%). Over half of the patients had cardiovascular disease (n = 160, 53.2%); the other comorbidities were malignancy in 22.6% (n = 68), dementia in 21.9% (n = 66), previous stroke in 17.3% (n = 52), and acute renal failure in 47.2% (n = 142). The median APACHE II score was 20.0 (IQR = 14.0–26.0, range 2–41). The median GCS was 15.0 (IQR = 12.0–15.0, range 3–15). The dopamine dose was moderate in most patients (n = 201, 66.8%); low in 23.3% (n = 70); and high in 10.0% (n = 30).

**Table 1:** Demographic and clinical characteristics of patients (N = 301).

Variable	Overall
**Age (years)**	
Median (IQR)	66.0 (55.0–79.0)
Range	18–104
**Age (years), n (%)**	
<60	106 (35.2)
≥60	195 (64.8)
**Gender, n (%)**	
Male	171 (56.8)
Female	130 (43.2)
**Setting of care, n (%)**	
ICU	164 (54.5)
IM	137 (45.5)
**Cardiovascular disease, n (%)**	
No	141 (46.8)
Yes	160 (53.2)
**Malignancy, n (%)**	
No	233 (77.4)
Yes	68 (22.6)
**Dementia, n (%)**	
No	235 (78.1)
Yes	66 (21.9)
**History of previous stroke, n (%)**	
No	249 (82.7)
Yes	52 (17.3)
**Acute renal failure, n (%)**	
No Yes	159 (52.8) 142 (47.2)
**APACHE II score**	
Median (IQR)	20.0 (14.0–26.0)
Range	2–41
**Glasgow coma scale**	
Median (IQR)	15.0 (12.0–15.0)
Range	3–15
**Average dopamine dose (µg/kg/min), n (%)**	
Low (<5)	70 (23.3)
Moderate (5–10)	201 (66.8)
High (>10)	30 (10.0)

APACHE II, acute physiology and chronic health evaluation; ICU, intensive care unit; IM, internal medicine; IQR, interquartile range; µg/kg/min, micrograms per kilograms per minute

### Demographic and clinical characteristics according to patient GOCs

**Fig 1:**
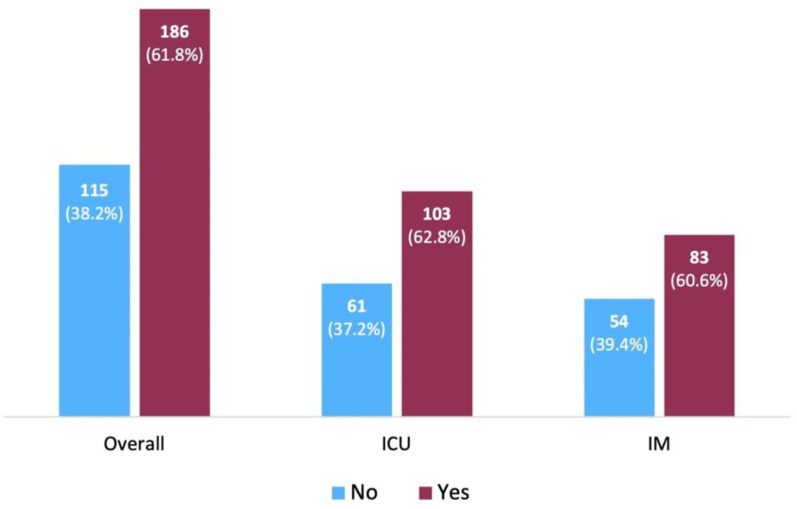
Comparison of patient goals of care discussion (“No” vs. “Yes”), overall and by care setting: internal medicine (IM) and intensive care unit (ICU).

(Fig 1) illustrates the distribution of patients according to the presence or absence of GOC discussions. Overall, 61.8% of the patients (n = 186) had their GOCs discussed. The proportion of patients with documented GOC discussions was similar between the ICU (62.8%, n = 103) and IM ward (60.6%, n = 83) settings. Compared with the non-discussed GOC group, the discussed GOC group was significantly older (median age: 68 years, IQR = 58–81 years vs. 60 years, IQR = 51–72, P < 0.001); comprised a significantly larger proportion of patients aged ≥60 years (n = 136, 73.1% vs. n = 59, 51.3%, p < 0.001) and those with dementia (n = 49, 26.3% vs. n = 17, 14.8%, p = 0.018); had significantly higher median APACHE II score (21.0, IQR = 15.0–27.0 vs. 18.0, IQR = 11.5–23.5, p = 0.001); and had significantly lower GCS (15.0, IQR = 11.0–15.0 vs. 15.0, IQR = 15.0–15.0, p < 0.001) (Table 2). Gender, cardiovascular disease, malignancy, history of previous stroke, acute renal failure, and dopamine dose were not significantly different between the groups. Notably, while GOC discussion rates were similar between ICU and IM patients, IM ward participants exhibited markedly greater disease severity, evidenced by a higher median APACHE II score (22.0 [IQR: 17.0–28.0] versus 18.0 [IQR: 12.0–22.0]; P < 0.001), and a poorer neurological status (median Glasgow Coma Scale 14.0 [IQR: 10.0–15.0] versus 15.0 [IQR: 15.0–15.0]; P < 0.001). Dementia was more prevalent in IM patients compared to ICU patients (35.0% vs. 11.0%; P < 0.001), although cardiovascular disease was more common in ICU patients (59.1% vs. 46.0%; P = 0.023). These findings indicate that, despite IM ward patients exhibiting increased illness severity, the frequency of GOC conversations was similar across settings. (S1 Table).

**Table 2:** Comparison of demographic and clinical characteristics of patients by goal of care discussion (N = 301).

Variable	Patients’ GOC discussed *(n = 186, 61.8%)*	Patients’ GOC not discussed *(n = 115, 38.2%)*	P-value
**Age (years)**			**<0.001**
Median (IQR)	68.0 (58.0–81.0)	60.0 (51.0–72.0)	
**Age (years), n (%)**			**<0.001**
<60	50 (26.9)	56 (48.7)	
≥60	136 (73.1)	59 (51.3)	
**Gender, n (%)**			0.523
Male	103 (55.4)	68 (59.1)	
Female	83 (44.6)	47 (40.9)	
**Setting of care, n (%)**			0.693
ICU	103 (55.4)	61 (53.0)	
IM	83 (44.6)	54 (47.0)	
**Cardiovascular disease, n (%)**			0.836
No	88 (47.3)	53 (46.1)	
Yes	98 (52.7)	62 (53.9)	
**Malignancy, n (%)** No Yes	138 (74.2) 48 (25.8)	95 (82.6) 20 (17.4)	0.090
**Dementia, n (%)**			**0.018**
No	137 (73.7)	98 (85.2)	
Yes	49 (26.3)	17 (14.8)	
**History of previous stroke, n (%)**			0.326
No	157 (84.4)	92 (80.0)	
Yes	29 (15.6)	23 (20.0)	
**Acute renal failure, n (%)** No	94 (50.5)	65 (56.5)	0.312
Yes	92 (49.5)	50 (43.5)	
**APACHE II score**			**0.001**
Median (IQR)	21.0 (15.0–27.0)	18.0 (11.5–23.5)	
**Glasgow coma scale**			**<0.001**
Median (IQR)	15.0 (11.0–15.0)	15.0 (15.0–15.0)	
**Average dopamine dose, (µg/kg/min), n (%)**			0.298
Low (<5)	38 (20.4)	32 (27.8)	
Moderate (5–10)	130 (69.9)	71 (61.7)	
High (>10)	18 (9.7)	12 (10.4)	

GOC, goals of care; APACHE II, acute physiology and chronic health evaluation; ICU, intensive care unit; IM, internal medicine; IQR, interquartile range; µg/kg/min, micrograms per kilograms per minute

### Regression analysis of factors associated with patient GOCs

On multivariable logistic regression analysis (Table 3), age ≥ 60 years was independently associated with the presence of GOC discussion (OR 2.408, 95% CI 1.341–4.321, p = 0.003). Other factors, such as dementia, APACHE II score, and GCS, which were found to have significant associations with GOC discussions in the univariable analysis, did not remain significant in the multivariable analysis.

**Table 3:** Univariable and multivariable logistic regression model for variables associated with patients goal of care.

Variable	Univariable analysis Odds Ratio (95% CI)	P-value	Multivariable analysis Odds Ratio (95% CI)	P-value
**Age** (≥60 years old)	2.582 (1.584–4.208)	**<0.001**	2.408 (1.341–4.321)	**0.003**
**Gender** (Female)	1.166 (0.728–1.867)	0.523	–	–
**Setting of care** (IM)	0.910 (0.571–1.451)	0.693	–	–
**Cardiovascular disease**	0.952 (0.597–1.517)	0.836	–	–
**Malignancy**	1.652 (0.922–2.961)	0.092	–	–
**Dementia**	2.062 (1.121–3.793)	**0.020**	1.044 (0.512–2.131)	0.905
**History of previous stroke**	0.739 (0.404–1.353)	0.327	–	–
**Acute renal failure**	1.272 (0.797–2.031)	0.313	–	–
**APACHE II score**	1.050 (1.020–1.081)	**0.001**	1.016 (0.981–1.052)	0.379
**Glasgow coma scale**	0.886 (0.811–0.968)	**0.007**	0.903 (0.815–1.000)	0.050
**Average dopamine dose average** (µg/kg/min) Low (<5) Moderate (5–10) High (>10)	Reference 1.542 (0.888–2.678) 1.263 (0.530–3.011)	0.300	–	–

APACHE II, acute physiology and chronic health evaluation; CI, confidence interval; IM, internal medicine; IQR, interquartile range; µg/kg/min, micrograms per kilograms per minute

### Survival analysis results

**Fig 2:**
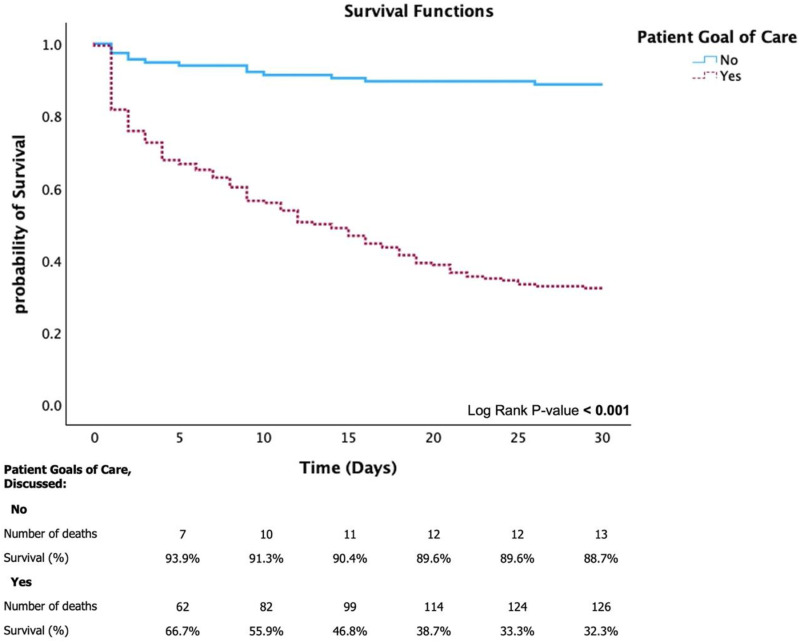
Kaplan–Meier survival curve for 30-day mortality adjusted by patient goals of care.

**Fig 3:**
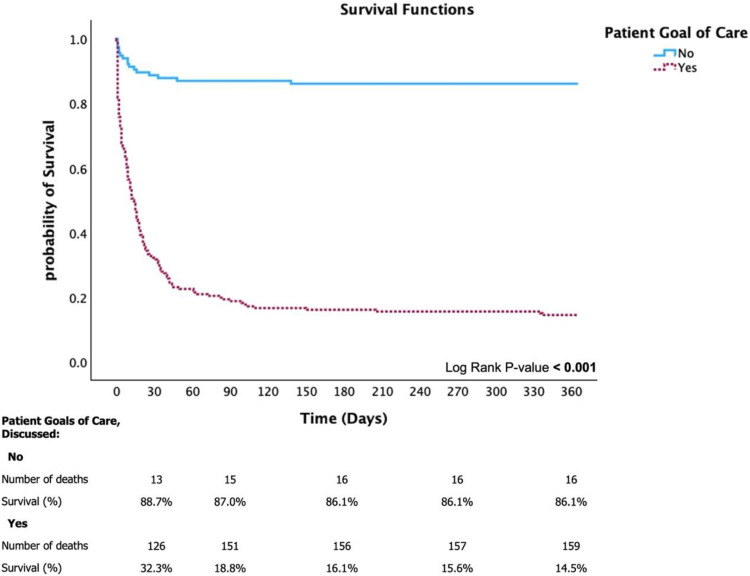
Kaplan–Meier survival curve for 1-year mortality adjusted by patient goals of care.

Kaplan–Meier analysis (Fig 2) revealed significantly higher survival rates in the non-discussed GOC group than in the discussed GOC group at 30 days (88.7% vs. 32.3%) (log-rank p < 0.001). The results indicated a decline in survival rates over time among patients with documented GOC discussions. For 1-year survival, the survival rates at 90 days and 1 year were 87.0% and 86.1%, respectively, in the non-discussed GOC group and 18.8% and 14.5%, respectively, in the discussed GOC group (Fig 3).

## Discussion

In this study, we found that GOC discussions were not widely implemented, even among critically ill patients who require vasopressors, such as dopamine. Importantly, approximately 60% of the cohort in both the ICU and IM wards had recorded GOC discussions, regardless of disease severity. GOC discussions are crucial for aligning medical interventions with the values and preferences of critically ill patients. In Saudi Arabia, a recent study on critically ill patients showed that only 13% of the cohort had DNR orders, of which the predictors were older age, higher APACHE II scores, and poor functional status [30]. Similarly, as shown in our study, older age was an independent predictor of GOC discussions as indicated in other studies of advance care planning and GOC engagement. Sharp et al. [31] have recently showed that frailty was a robust indicator of GOC recommendations, and it was an important indicator of proactive discussions among the elderly population. Uyeda et al.[32] also demonstrated that older age and increased disease burden were significant predictors of GOC discussions. Aldawood et al. [33] reported that 77% of ICU deaths were preceded by end-of-life decisions; among these, 66% gave DNR directives. The findings of this study corroborated those of international estimates of GOC discussions. In the United States, Fleming et al [6] demonstrated significant gaps in shared decision-making among critically ill neurological patients, with only <10% of meetings reporting comprehensive shared decision-making. Another study in the United States showed a 36.4% prevalence of GOC documentation among hospitalized patients, particularly within the first 24 h of admission. However, Orford et al revealed disparities among racial and ethnic minorities; in particular, only 34.4% of patients in Australia who had life-threatening illnesses had documented GOC conversations [9]. Taken together, GOC discussions remain to have a suboptimal prevalence and often occur only when patients are acutely or critically ill.

Our study demonstrated that advanced age was significantly associated with documented GOC discussions, patients aged over 60 years were more than twice as likely to have a documented GOC discussion compared to younger individuals. Similarly, international trends showed that older and critically ill patients were often prioritized for advanced care conversations [34–36]. This finding aligns with the results of other studies showing an association between older age and a relatively high likelihood of initiating conversations about care goals [37]. However, several older adults lack decision-making capacity when acutely ill, and attempting to establish a patient’s GOC at this point becomes a considerable challenge [38]. Understanding patients’ attitudes can significantly shape their treatment plans and potentially improve health-related outcomes; this highlights the importance of adopting a tailored approach when providing care for older adults [39–41]. However, the low frequency of GOC discussions among younger patients indicates missed opportunities to involve them in important care planning, address unpredictable complications, and elucidate treatment preferences. Involving younger patients in these discussions, despite their perceived lower risk, could improve the quality of care and ensure that treatment is congruent with their values and goals [42–44].

In addition to age, GOC discussions were significantly higher among patients with poor GCS and dementia. This probably indicates clinicians’ awareness of unfavorable prognoses and limited opportunity for functional recovery in these groups. Evidence has shown that mortality was associated with lower GSC scores and worse neurological outcomes [45]. While dementia is commonly associated with progressive frailty and declining functional status—both key triggers for initiating GOC discussions and considering palliative approaches [46,47]. The higher prevalence of GOC documentation in these subgroups in our study suggests that clinicians are responding to clinical markers of deterioration. However, the variability in timing and consistency of these conversations underscores the need for clearer protocols to support timely, patient-centered decision-making.

Notably, high APACHE II scores differed significantly between patients with and without documented GOC discussions. High APACHE II scores, which typically indicate a more critical condition, may prompt healthcare teams to urgently prioritize patient preferences [48,49]. This finding reflects a reactive approach, in which GOC discussions are primarily focused on critically ill patients who clearly have poor prognosis. However, the timing of GOC discussions is crucial [50,51]. Although the exact timing of each GOC discussion was not a primary objective, our findings showed that these discussions occurred later during admission, where severe illness markers are apparent. A pooled analysis of 12 eligible articles highlighted significant variations in the timing of these discussions and concluded that discussions that were initiated earlier, particularly at the time of facility admission, were associated with more favorable outcomes, such as care aligned with patient preferences and reduction of unnecessary interventions [50]. Similarly, a retrospective cohort study found that GOC discussions were more likely to be accepted by patients who were terminally ill and those with organ failure and frailty [52]. Despite the greater severity of illness and poorer neurological status among IM ward participants, the prevalence of documented GOC discussions was similar to that of ICU participants. This may indicate systemic or cultural barriers to the initiation of GOC outside critical care settings, a gap in standardized clinical triggers for discussion, or heightened uncertainty concerning prognosis in non-ICU environments. The observed discrepancies indicate that critical illness may not reliably trigger timely GOC discussions, especially in general ward settings. This highlights the potential necessity for institutional protocols to promote more equitable and proactive engagement practices. These findings underscored the pressing need for healthcare professionals to adopt GOC discussions as proactive standard practice rather than delay them until patients are critically or terminally ill. Early and consistent conversations about GOCs can better align treatment plans with patient values, ultimately enhancing the quality of care at the end of life [53–55].

Based on the above observations, several proactive approaches may be implemented to improve the timing and frequency of GOC conversations. First, institutions can ensure early GOC practices by assuring the integration of discussions into standard admission protocols for populations at risk, such as those aged more than 60 years or those requiring vasopressors. This is in line with the previous suggestions of adopting early discussions to reduce unwanted interventions and improvement alignment of care [50,56]. Second, automatic alerts can be established based on specific triggers of severity scoring criteria, such as APACHE II ≥ 20 or a rapid decline in GCS. These severity-based alerts would be suggestive of further multidisciplinary team reviews to initiate GOC discussions, and the reliance on such scoring systems has proved beneficial in early palliative care engagement [48,57]. Third, GOC discussions can be standardized by embedding those conversations into electronic medical record systems, including prompts upon ICU transfer or vasopressor initiation. This way, these strategies can help shift GOC conversations from a reactive approach to proactive, value-centered discussions that improve patient-oriented care, enhance communication and reduce distress among healthcare providers. Additionally, encouraging GOC discussions fosters more equitable and effective use of healthcare resources, especially for vulnerable groups with complex needs, affirms patient rights are upheld, and preserves ethical principles of beneficence and autonomy.

The study found significantly higher mortality rates at both 30 days and 1 year among patients with documented GOC discussions compared to those without. The importance of implementing thorough GOC discussions for critically ill patients at imminent risk of mortality cannot be overstated. However, this approach may reflect a tendency to reserve GOC discussions for patients in the most critical conditions, potentially highlighting disparities in the quality of GOC provided to critically ill patients at different stages of their illness [58]. Shared decision-making on dopamine initiation should be clearly documented and guided by information that carefully weighs the perceived risks and benefits. These discussions are essential to ensure patient-centered care, fostering transparency and alignment of treatment strategies with the values and preferences of critically ill patients.

In Saudi Arabia’s cultural setting, end-of-life decision-making is influenced by Islamic bioethical norms that often discourage the use of interventions unlikely to yield meaningful enhancements in quality of life or clinical outcomes [59]. This provides a broad ethical framework that supports GOC conversations and aligns clinical decision-making with patients’ values and religious principles [20]. Family members act as surrogate decision-makers for older adults and incapacitated patients, which can limit the direct participation of patients in GOC conversations. Furthermore, physicians may be hesitant to initiate effective end-of-life communications due to fears of legal repercussions, emotional distress or cultural limitations surrounding death discussions [18]. Even if the religious-based decisions support the cessation of non-beneficial treatments, there may be inconsistencies in the application of those decisions into clinical behavior. Indeed, previous studies have emphasized the need for improved communication and cultural competency among healthcare providers to better implement GOC conversations [33]. However, despite its established importance, adoption of GOC discussions remains inconsistent, especially in regions with institutional and cultural challenges [54]. Clinical communication and cultural competence of clinicians can be improved through dedicated training programs and institutional support to align GOC practices with ethical and religious expectations [19].

It is noteworthy that the higher mortality observed among patients with documented GOC discussions may reflect a documentation bias rather than a causal relationship. Clinicians often initiate and record GOC conversations among patients with advanced disease and those with poor prognosis as indicated by high APACHE II scores, reduced consciousness or end-stage comorbidities.

This may have led to selective documentation practices, whereby GOC discussions are more likely to be recorded in patients already at high risk of death. This interpretation aligns with previous studies, which have shown that end-of-life discussions are most commonly documented among patients with terminal trajectories or those admitted to palliative care settings[49,60]. Therefore, the current findings on mortality should be interpreted with caution, as they likely reflect clinical decision-making practices that prioritize GOC conversations for the most critically ill—rather than indicating that GOC discussions contribute to increased mortality.

This study had several strengths, including the comprehensive analysis of a substantial patient cohort who received dopamine, providing significant insights into the patterns of GOC discussions and their association with patient outcomes, particularly those with high morbidity and mortality risks. Furthermore, this study filled a gap in the literature on GOC practices in the Middle East, where cultural and religious factors are significant drivers of end-of-life care. The robust methodological approach and multivariable analysis ensured reliable identification of independent relationships and survival outcomes.

However, there were few limitations in this study. The retrospective study design inherently limited the ability to infer causality due to possible selection and documentation biases. Next is the single-center setting in a tertiary hospital, which may restrict the generalizability of the findings to other institutions with different practices and policies. In addition, differences in patient health status and clinical practices between the ICU and IM ward settings may have introduced confounding factors. Moreover, we did not account for the concurrent use of vasopressors other than dopamine or supportive measures and we did not have data on the dopamine administration route (e.g., central vs. peripheral venous lines) for patients in the ICU; these data could have provided a more in-depth understanding of treatment outcomes across different settings. Additionally, due to the fact that the current study relied on chart review, GOC discussions are presumably under-documented, because these conversations might have occurred informally but were not recorded. This might have influenced the overall prevalence rate. Moreover, the findings of the current study might not be generalizable to other healthcare systems, particularly those with different cultural, religious or institutional norms in terms of end-of-life care. To address these limitations and better account for these variations, future research could stratify analyses to accurately reflect differences among care settings and include more detailed data on patient characteristics, such as clinical interventions, illness trajectories, and baseline prognoses. Moreover, prospective and multicenter designs are essential to address these knowledge gaps, and specific strategies should be implemented to ensure adequate documentation of GOC conversations.

## Conclusions

This study provided important insights into the patterns and associations of GOC discussions among hemodynamically unstable critically ill patients in Saudi Arabia. Although advanced age and higher illness severity were correlated with a greater likelihood of GOC discussions, the timing and implementation of these discussions remain inadequate. Overcoming institutional and cultural obstacles to promote comprehensive, early, and proactive GOC discussions is crucial for enhancing patient-centered care and aligning treatment approaches with patient values and preferences. The observed association between documented GOC discussions and higher mortality highlights a reactive pattern in which conversations are initiated late in the clinical course. Institutions should implement early initiation protocols, culturally-sensitive communication frameworks, and standardized clinical triggers to facilitate timely GOC discussions throughout the continuum of care, thereby promoting a proactive and patient-centered approach.

## Supporting information

S1 Table Comparison of demographic and clinical characteristics of patients by care setting (N  = 301). (DOCX)
