# Supplementary material for: Goals of care discussions among critically Ill patients on vasopressor treatment
Source: PLoS One. 2025 May 28;20(5):e0324482. doi: 10.1371/journal.pone.0324482 (PMC12119013; doi:10.1371/journal.pone.0324482)
Supplement: S1 Table — (DOCX) [file pone.0324482.s001.docx]

**Table Legends**

**S1 Table.** Comparison of demographic and clinical characteristics of patients by care setting (N = 301).

| **Variable** | **ICU**  *(n = 164, 54.5%)* | **IM**  *(n = 137, 45.5%)* | **P-value** |
| --- | --- | --- | --- |
| **Age (years)**  Median (IQR) | 64.0 (56.0 – 71.0) | 74.0 (54.0 – 83.0) | **<0.001** |
| **Age (years), n (%)**  <60  ≥60 | 65 (39.6)  99 (60.4) | 41 (29.9)  96 (70.1) | 0.079 |
| **Gender, n (%)**  Male  Female | 96 (58.5)  68 (41.5) | 75 (54.7)  62 (45.3) | 0.508 |
| **Patients’ GOC, n (%)**  Not discussed  Discussed | 61 (37.2)  103 (62.8) | 54 (39.4)  83 (60.6) | 0.693 |
| **Cardiovascular disease, n (%)**  No  Yes | 67 (40.9)  97 (59.1) | 74 (54.0)  63 (46.0) | **0.023** |
| **Malignancy, n (%)**  No  Yes | 132 (80.5)  32 (19.5) | 101 (73.7)  36 (26.3) | 0.162 |
| **Dementia, n (%)**  No  Yes | 146 (89.0)  18 (11.0) | 89 (65.0)  48 (35.0) | **<0.001** |
| **History of previous stroke, n (%)**  No  Yes | 141 (86.0)  23 (14.0) | 108 (78.8)  29 (21.2) | 0.103 |
| **Acute renal failure, n (%)**  No  Yes | 86 (52.4)  78 (47.6) | 73 (53.3)  64 (46.7) | 0.884 |
| **APACHE II score**  Median (IQR) | 18.0 (12.0 – 22.0) | 22.0 (17.0 – 28.0) | **<0.001** |
| **Glasgow coma scale**  Median (IQR) | 15.0 (15.0 – 15.0) | 14.0 (10.0 – 15.0) | **<0.001** |
| **Average dopamine dose, (µg/kg/min), n (%)**  Low (<5)  Moderate (5–10)  High (>10) | 48 (29.3)  99 (60.4)  17 (10.4) | 22 (16.1)  102 (74.5)  13 (9.5) | **0.019** |

GOC, goals of care; APACHE II, acute physiology and chronic health evaluation; ICU, intensive care unit; IM, internal medicine; IQR, interquartile range; µg/kg/min, micrograms per kilograms per minute
